# Science, evidence, law, and justice

**DOI:** 10.1073/pnas.2312529120

**Published:** 2023-10-02

**Authors:** Thomas D. Albright, David Baltimore, Anne-Marie Mazza, Jennifer L. Mnookin, David S. Tatel

**Affiliations:** ^a^The Salk Institute for Biological Studies, La Jolla, CA 92037; ^b^California Institute of Technology, Pasadena, CA 91125; ^c^The National Academies of Sciences, Engineering, and Medicine, Washington, DC 20001; ^d^University of Wisconsin, Madison, WI 53706; ^e^United States Court of Appeals for the District of Columbia Circuit, Washington, DC 20001

**Keywords:** Committee on Science, Technology, and Law, pattern comparison, rules of evidence, forensic reform

## Abstract

For nearly 25 y, the Committee on Science, Technology, and Law (CSTL), of the National Academies of Sciences, Engineering, and Medicine, has brought together distinguished members of the science and law communities to stimulate discussions that would lead to a better understanding of the role of science in legal decisions and government policies and to a better understanding of the legal and regulatory frameworks that govern the conduct of science. Under the leadership of recent CSTL co-chairs David Baltimore and David Tatel, and CSTL director Anne-Marie Mazza, the committee has overseen many interdisciplinary discussions and workshops, such as the international summits on human genome editing and the science of implicit bias, and has delivered advisory consensus reports focusing on topics of broad societal importance, such as dual use research in the life sciences, voting systems, and advances in neural science research using organoids and chimeras. One of the most influential CSTL activities concerns the use of forensic evidence by law enforcement and the courts, with emphasis on the scientific validity of forensic methods and the role of forensic testimony in bringing about justice. As coeditors of this Special Feature, CSTL alumni Tom Albright and Jennifer Mnookin have recruited articles at the intersection of science and law that reveal an emerging scientific revolution of forensic practice, which we hope will engage a broad community of scientists, legal scholars, and members of the public with interest in science-based legal policy and justice reform.

The scientific enterprise is often recognized as the most powerful contributor to our collective knowledge and well-being, propelling discoveries, guiding difficult decisions, and generating transformative instruments and techniques. While the advances of science thus create remarkable new opportunities for understanding and engaging with the natural world, it is equally obvious that the forward march of science produces challenges as well as benefits. A widely promoted scientific invention may not be safe, or its use may sacrifice civil liberties, or have disparate impact on different segments of the population. Real or perceived benefits of an invention may, nonetheless, lead to pressure for rapid deployment before risks have been adequately assessed and mitigated. Consider, for example, as just three instances of many, the recent meteoric rise of algorithms for assessing person identity from facial images, or rapidly developing large language model AIs, or our ability to edit genetic code. In each case, widespread deployment may well occur before the risks and benefits have been carefully assessed or any thoughtful regulatory structure created to guide its operation.

This disconnect between scientific advances and legal policy development is hardly new. The development of a comprehensive regulatory framework for drug safety and efficacy lagged decades behind the fast-moving science of drug development (1–3). Physician-entrepreneurs of the 1940s and 1950s removed large parts of the human brain as treatment for mental health disorders, based on dubious theory, no meaningful validation, and little government oversight (4, 5). Today, we routinely witness promotions of self-driving cars and tools for cognitive enhancement in a society that possesses limited understanding of the efficacy, risks, and liabilities of emerging technologies. Conversely, while our courts and legislatures promote carefully worded standards to guard against “junk science” in litigation, both the scientific community and much of the legal community recognize that these standards, in practice, are often leaky, ineffective, and inadequate as a check on the use of shoddy science in the legal system.

## The Committee on Science, Technology, and Law

The foregoing examples highlight the need for a closer intellectual partnership between the disciplines of science and law. To that end, and in partial response to recent Supreme Court decisions on scientific evidence (6–8), the leadership of the National Academy of Sciences (NAS) entered into discussions in the late 1990 s about establishing a working group for this purpose. The creation of a standing committee within the National Academies devoted to issues at the interface of science and law was not an easy decision. Many scientists within the National Academies viewed the sometimes brutal adversarial nature of the courtroom, and legal culture more generally, as an unsuitable focus for an institution devoted to the rigorous scholarly search for scientific truth. Nonetheless, the need for a prominent forum for representatives of these communities to get to know each other, understand each others’ cultures, and exchange ideas was becoming more and more evident.

In March 2000, Donald Kennedy and Richard Merrill convened the Committee on Science, Technology, and Law (CSTL), a new standing committee under the auspices of the National Academies of Sciences, Engineering, and Medicine. Kennedy and Merrill sought to bring together distinguished members of the science and law communities to stimulate discussions that would lead to a better understanding of the role of science in legal decisions and government policies and to a better understanding of the legal and regulatory frameworks that govern the conduct of science. At biannual meetings, scientists and members of the legal community, including members of the legal academy and judiciary, were encouraged to bring to the committee topics of national importance that would be best addressed from the perspective of both communities. Sessions at each meeting were built around controversial or emerging issues and often led to the development of project ideas for consensus studies and convening activities.

At the time it was established, Kennedy and Merrill noted that CSTL could “not hope to canvass the entire terrain. Instead, we hope to become one of several contributors to the growing dialogue between science, engineering, and law; a supporter of initiatives by other organizations; and a catalyst for promoting productive collaboration among participants from all affected disciplines.” Nearly 25 y later, it is probably fair to say that Kennedy and Merrill could never have envisaged either the wide range of topics that CSTL would explore or the impact of these explorations.

In 2009, Kennedy and Merrill passed leadership of CSTL to Richard Meserve and David Korn, and in 2015, Meserve and Korn passed leadership of the committee to David Baltimore and David Tatel (coauthors of this *Introduction*). In 2023, the baton was passed again to Martha Minow and Harold Varmus. And with hindsight, it is clear that the National Academies’ and Kennedy and Merrill’s decision to establish CSTL was prescient.

Many areas of intersection between the disciplines of science and law involve developing, regulating, preventing, or promoting activities that have broad societal impact. Scientific knowledge is commonly used, for example, to guide regulation of environmental policy, medical practice, energy production, transportation safety, economic welfare, education, security, and defense. Conversely, law is often used to control applications of science, and technology born from it, that may compromise safety or individual freedoms. CSTL has ventured into a substantial set of these domains, with discussions, workshops, and reports focusing on diverse topics such as genome editing, climate intervention, implicit racial bias, disinformation in social media, synthetic biology, and voting systems.

## Science, Law, and Forensics

One of the largest footprints of CSTL—and arguably the most impactful of all science-law engagements today—is forensics.^*^ In most legal contexts, forensic practices seek evidence of cause and responsibility for past actions that may be criminal and/or may have caused or produced loss or harm to others. Methods for doing so involve comparison of artifacts (e.g., a bullet shell casing) found at a particular location with a model or specimen (a shell casing from a specific gun) inspired by a hypothesis about the source of the artifacts (such as a suspect’s criminal activity). The comparison yields a classification decision (“inclusion” vs. “exclusion”, or “match” vs. “nonmatch”) based on a criterion level of similarity between the objects of comparison. A conclusion of inclusion or match supports the underlying hypothesis about source and may justify further investigation as well as criminal or civil action. For some forensic methods, such as those used for DNA or latent fingerprint examination, a match may even be deemed sufficient evidence, standing alone, to support a conviction.

**Fig. 1.**
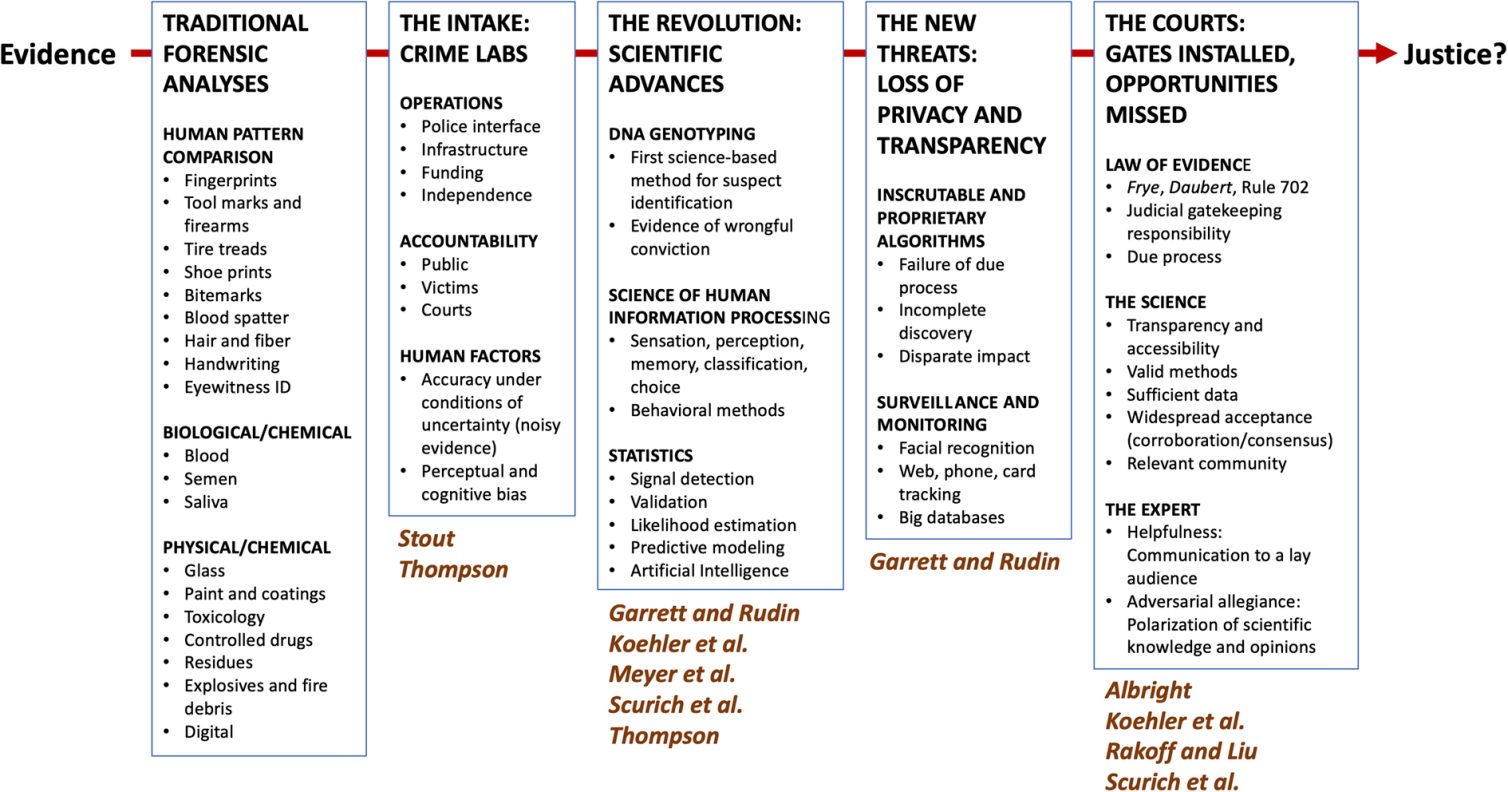
Overview of topics covered in this *PNAS* Special Feature Issue on Science, Evidence, Law, and Justice. The leftmost panel contains a taxonomy of traditional forensic subdisciplines. The remaining panels correspond to themes addressed by the articles in this collection. Corresponding authors are indicated below the themes (some articles overlap multiple themes).

There are many different forensic subdisciplines practiced today that follow this same general strategy, which can be taxonomized based on the types of media and measurement tools employed (Fig. 1, *Left*). Some involve instrument-based measurements (e.g., chromatography) and comparisons of physical, chemical, or biological substances. For example, the chemical components of a paint scraping found on a stone wall (artifact) may be assessed and compared with paint from a suspect’s car (model). Although interesting questions sometimes arise about precision of these methods, the underlying measurements, comparison processes, and criteria for classification are transparent and easily interrogated, and the operating principles are readily interpretable. All of this information can be used to predict decision accuracy.

Another common category of forensic analysis involves human measurement and comparison of visually patterned impressions, such as fingerprints and tool marks. Unlike instrument-based methods, human visual pattern comparison does not yield ready access to the underlying measure of similarity of artifact and model or to the criterion level of similarity used for the classification decision. In essence, a trained examiner looks at the comparison and determines whether the observed similarities provide an adequate basis for a match, but the standards an expert uses for such a judgment are currently neither statistical nor quantified. These methods simply provide the end result, which makes inferences about the accuracy of that decision difficult, if not impossible.^†^ The accuracy of a forensic conclusion, however, is what the court really needs to know.

Throughout much of the twentieth century, practitioners of forensic pattern comparison methods asserted that their accuracy was a function of their experience (9, 10):

*Court:* “What’s your error rate?”
*Forensic Witness:* “Zero.”
*Court:* “How can it be zero?”
*Forensic Witness:* “Well, in every case I’ve testified, the guy’s been convicted.”^‡^

Backed by the snowballing effect of legal precedent, and perhaps a limited appreciation of probability and statistics, claims of experience have routinely grounded the admission of forensic expert testimony in criminal trials. While few would deny the value of experience, it cannot substitute for empirical evidence of validity, assessed through carefully designed studies to determine the probability that a forensic method provides the correct answer (11). (Experienced psychics may well be no more accurate than inexperienced ones.) Spurred to action in the 1990 s by the Supreme Court’s *Daubert* ruling on the validity of scientific evidence (6), the rise and scientific scrutiny of DNA evidence (12), and the gut-wrenching impact of wrongful convictions (13)—the vast majority of whom are people of color—both scientific and legal communities took increasing notice of the problem of forensic validity.

Beginning in 2007, urged on by leaders in the forensic science community, an expert committee of scientists, statisticians, and legal professionals was convened by the National Academy of Sciences under the auspices of CSTL to perform a comprehensive evaluation of forensic practices in the United States. This congressionally mandated study, published in 2009 (10), identified significant weaknesses associated with validation, training, and reporting in forensic practice and included detailed recommendations for science-based reform.^§^ These recommendations led to creation of the short-lived National Commission on Forensic Science (an advisory body to the US Department of Justice) (14), the National Institute of Standards and Technology (NIST) operation known as the Organization of Scientific Area Committees for Forensic Science (OSAC), the Center for Statistics and Applications in Forensic Evidence (CSAFE), which supports NIST's efforts to advance the utility of statistical methods for forensic analysis, and to a variety of grass-roots efforts to improve and standardize forensic practice. In 2015, President Obama asked the President’s Council of Advisors on Science and Technology (PCAST) to further evaluate needs within the forensic science community, the product of which was a 2016 report that raised concerns and recommendations specific to human pattern comparison methods (15).

These scientific evaluations highlight three broad principles for forensic reform in the interest of justice:

(1) A forensic method to be used as a basis for fateful decisions must be empirically validated by means of careful studies using known source samples—samples for which the correct classification decision is known—to yield a robust quantitative measure of the method’s accuracy.
(2) Scientific research should be conducted to assess sensory, perceptual, and cognitive factors that create uncertainty and engender bias in the context of pattern comparison, thus limiting accuracy, and to identify ways of lessening those effects.
(3) Law enforcement and the courts—the end users of forensic conclusions—must be made aware of limits on accuracy and incorporate growing scientific knowledge that bears on the application of forensic testimony to the facts of a case at hand.

In the spirit of these principles—method validation, assessment and mitigation of accuracy-limiting factors, and education of the users of forensic testimony—we have commissioned eight articles to address the potential for science-based reform from several distinct vantage points. Contributing authors include cognitive, neural, and computer scientists, experimental psychologists, legal scholars and judges, and the director of a major city crime lab, all of whom have pioneered application of their professions to a convergence of science and law in the interest of justice. The stories told here explore myriad issues ranging from the messy, urgent, disturbing, and endlessly frustrating details of forensic evidence collection and analysis, to the carefully crafted but leaky rules by which that evidence contributes to justice. In the spaces in between, law professors and scientists promote normative standards and offer scientific insights that can improve courtroom decisions.

These articles comprise four overlapping themes (Fig. 1):

(1) The Intake: Operation of a crime lab, where forensic evidence enters the justice system and is subjected to a variety of analyses;
(2) The Revolution: Scientific advances that are now rewriting the script for forensic investigation;
(3) The New Threats: Risks to justice posed by new technologies that render decisions by invasive and inscrutable processes; and
(4) The Courts: Rules for use of scientific evidence by the courts and their varying interpretations by the judiciary.

## The Intake: Operation of a Crime Lab

In an ideal world, criminal investigation and prosecution build upon evidence that neatly conforms to the standards of modern science. Television crime shows notwithstanding, good forensic evidence is often hard to collect, difficult to keep track of, and harder to analyze. Peter Stout, director of the Houston Forensic Science Center (HFSC)—one of the largest crime labs in the country—opens this collection of articles with a marvelous first-person narrative of the challenges that arise from this type of operation (Fig. 1, second panel) (16). Under Stout’s leadership, HFSC rose from the ashes of a notorious public failure of justice (17), in which scientific principles and controlled practice were little to be seen (18), to become an exemplar of forensic science for the public good (19, 20). One of the most important features of this new crime lab is that it operates as an independent contributor^¶^ to a coherent, highly integrated, and scientifically grounded system of governance, law enforcement, forensic investigation, and judicial practice. All of these components aim to be accountable to numerous stakeholders, including the victims and their families.

Among many operating challenges, Stout relates the task of implementing “blind testing” of forensic examiners—a performance evaluation procedure in which examiners are tested using “fake” evidence for which the correct classification is known. Blind testing has been deemed imperative by academic analysts as a means to assess accuracy but has often been viewed by practitioners as being too difficult, too impracticable, or downright impossible to implement. Houston has taken on this challenge. The trick is to make this contrivance indistinguishable from real evidence, so that examiners bring the same expectations to the table and do not realize that they are being tested, which might influence their decisions. With tones of dark humor, Stout tells us that real evidence is often of such appallingly bad quality that it is difficult to imitate: “Evidence comes from the real world and will never be clean or designed to be reproducible like a research project. Odds are good that it is going to be decayed, smelly, sticky, foul and unusual.” While other articles in this collection highlight sensible and righteous strategies to improve the contributions of science to justice, Stout’s perspective anchors us first in the harsh, imperfect, costly, and frequently demoralizing world of forensic evidence: “Everything we do is the remains of someone’s worst day.”

### Contextual Bias in Forensic Examination.

For human pattern comparison disciplines, crime labs are where initial assessments of pattern similarity and classification decisions are made by forensic examiners (Fig. 1, second panel). Under conditions of uncertainty, these decisions are highly susceptible to contextual bias. For example, a fingerprint examiner may unconsciously lower their threshold for an inclusion decision after viewing photos of mutilated homicide victims (21). In recognition of this potential for bias, some crime labs have adopted strategies to restrict access to information that is not “task relevant” (22), such that, for example, the fingerprint examiner is not privy to any information about an investigation other than prints themselves. A persistent counterargument is that the additional information afforded by context contributes to more accurate decisions (23–25). Employing a Bayesian network model of the forensic decision process, Bill Thompson’s research article in this collection examines the impact of varying decision thresholds on probabilities of true and false convictions (26). Thompson’s thoughtful analysis proves that use of lower decision thresholds, induced by task-irrelevant information, can markedly increase—not decrease—the risk of convicting an innocent person. More generally, this analysis reveals that small changes in decision threshold can have a large impact on accuracy, which calls into question accuracy claims drawn from traditional nonblinded validation studies, where examiner expectations and decision thresholds may differ significantly from real forensic casework. This type of quantitative modeling focused on a specific question of practice is precisely what is needed to overcome longstanding but errant forensic logic and strategy.

## The Revolution: Wrongful Conviction, Empirical Frameworks, and the Science of Human Information Processing

“The debate and rigor of academic science is now influencing much of forensic science and that is the most significant change from the past” (27).

This recent quote from the National Institute of Justice captures the spirit of the scientific revolution that we are now witnessing in forensics. Many important scientific and technical developments (Fig. 1, third panel) have a) highlighted weaknesses and risks associated with forensic practice, b) drawn the attention of basic scientists with expertise in human decision-making and predictive modeling, and c) become poised to revolutionize forensic theory and practice.

### Exposing Wrongful Conviction.

Concerns about wrongful conviction date at least to the nineteenth century. At the same time, many legal professionals believed that real-world wrongful convictions for serious crimes were virtually nonexistent; the eminent Judge Learned Hand opined in 1923 that while the “ghost of the innocent man convicted” may haunt us, “it is an unreal dream” (28). In the 1980 s, however, following the invention of a chemical method known as the polymerase chain reaction (PCR), Learned Hand was proven wrong. PCR made it possible to amplify minute quantities of DNA recovered from a crime scene, such that the forensic genotype could be assessed and compared with that from people accused or convicted. This new forensic tool provided an independent and soundly science-based means to evaluate conclusions drawn from older forensic pattern comparison methods (as well as other forms of evidence, like eyewitness testimony and confessions). This process identified scores, and eventually hundreds, of prisoners who, it turns out, are not evidentially associated with biological material linked to the crime for which they had been convicted. This naturally casts significant doubt on the validity of the older forensic methods, many of which are still in common use and frequently still deemed admissible as evidence in litigation. It also triggered increased concerns about the accuracy of other forms of evidence ranging from eyewitness identification to jailhouse snitches.

### Establishing an Empirical Framework.

In their article for this collection (29), Nick Scurich, David Faigman, and Tom Albright stress the importance of adopting a sound empirical framework. Human pattern comparison disciplines have long lacked such a framework. Methods in common use today were originally conceived to improve law enforcement, and they were embraced because it seemed eminently plausible that people could compare and judge the similarity of things they observe. After all, we visually compare things all the time. We locate our car in the parking lot, we choose the ripest piece of fruit in the basket, or we identify our friend in the crowd. We might also instantly recognize our mother’s voice on the telephone or identify our spouse’s handwriting. However useful these abilities may be in everyday life, limits to the accuracy of our judgments are not typically tested, nor is our performance in any way scientific or based on explicit quantitative standards. In the past (and sometimes this practice, unfortunately, continues), forensic examiners would feint at scientific credibility by asserting that their opinions about the similarity of two visual patterns were accurate “to a reasonable degree of scientific certainty,”^#^ which carries about as much quantitative weight and probative value in the pattern vision domain as the assertion that the “rug really tied the room together” (30).

If we are to make comparative judgments that have real value for legal decisions, then performance standards must be sought within a well-defined empirical framework, rooted in the scientific method. This includes defining hypotheses and empirical questions, such as the following: What is the minimum discriminable difference for a given pattern type? What is the accuracy of the method used for discrimination? What are causes of error? It also includes a) employing suitable research methods and designing well-controlled experiments that yield reliable answers to these questions, and b) use of appropriate statistical tools and models, such that answers can be reported and conclusions can be drawn with known degrees of certainty. Forensic practice is in the still-early stages of developing an empirical framework of this sort, which has opened the field to evaluation from a perspective based in the modern sciences of human information processing.

### How People Make Decisions Based on Sensory Information.

Forensic patterns contain information, meaning that pattern comparison disciplines are necessarily dependent upon human brain systems for information processing, which include sensation, perception, memory, categorization, and choice. The science of these human information processing systems has grown by leaps and bounds. Much is now known about the operating characteristics of these systems, which reveal human aptitudes and weaknesses on tasks that rely upon stimulus detection, discrimination, selective attention, memory retrieval, and object recognition. Operating without this knowledge, as most human pattern matching disciplines have done for decades, is analogous to operating a mass spectrometer for chemical analysis of forensic samples without a user manual.^||^

These advances in sciences of human information processing have been complemented by improvements in the use and sophistication of statistical tools, including the application of principles from signal detection theory to evaluate decisions made by eyewitnesses (31, 32) and trained forensic examiners (33–35). These approaches suggest new behavioral and cognitive strategies for retrieving memories, limiting opportunities for bias, and improving decision-making by human observers. They also offer means to identify and precisely assess specific factors that influence examiner performance. Tools for rigorous predictive modeling—Bayesian inference, multivariate regression, and neural networks—have also entered the field with much promise, as illustrated, for example, by Thompson’s research article in this collection (26), highlighted above. These powerful tools also pose considerable risk, as Brandon Garrett and Cynthia Rudin argue in their essay for this collection (see below) (36).

### The Vanguard of Reform.

Jay Koehler, Jennifer Mnookin, and Michael Saks, a team of psychologists and legal scholars, offer a perspective on the scientific reinvention of forensics and present a coherent vision—and an emerging reality—of forensics either rewritten as sound science or cast aside (37). This reinvention consists, in part, of a shift of emphasis from the attributes of the expert conveying scientific testimony to the underlying scientific knowledge as it bears on the question before the court. As Tom Albright notes in a separate piece highlighted below (38), there has been a fair amount of waffling about the relative importance of scientific knowledge vs. the expert in the historical development of rules for the use of scientific evidence in litigation. Koehler et al. demonstrate a critical evolution along these lines among the users of forensic testimony—primarily the courts—from a “trust the examiner” zeitgeist to a “trust the scientific method” approach.

The perspective from Koehler et al. also offers a rich summary of scientific and legal policy ideas that have been aired in the reformist community in recent years and in some cases have become state of the art. Many of these ideas are rooted in key scientific advances and include a) improvements in methods for validation of forensic tools, such that courts can receive credible estimates of the accuracy of those tools when presented as the basis of scientific evidence in litigation; b) procedural reforms to reduce the influence of bias in the judgment and interpretation of forensic evidence; and c) a new reckoning of the longstanding but decidedly unscientific categorical approach to forensic conclusions and reporting (e.g., “it’s a match!”). On many of these topics, Koehler et al. go beyond characterization of the problems; they offer valuable suggestions for improvement.

These authors also highlight important, albeit incremental, policy proposals from advisory bodies, such as the NIST Organization of Scientific Area Committees for Forensic Science. But in the end, Koehler et al. stress that the big remaining problem lies with the courts: “if judges took seriously their duties under the *Daubert* line of cases (and state equivalents) and refused to admit insufficiently validated claims, the forensic sciences would adopt scientific practices more quickly and completely. Unfortunately, few courts have been so bold.” This abdication of gatekeeping responsibility by the courts is a recurring theme of articles in this collection, which we highlight below in a broader discussion of cause and resolution.

It is worth noting here that many other applied sciences that rely upon accurate high-stakes decisions in the face of sensory uncertainty and pressure of time, such as identifying tumors or weapons in radiographic images (39–41), prediction of severe weather events (42), or flying high-performance jet planes (43, 44), have successfully undergone scientific reinvention. So too must forensic practice continue down this emerging, if faltering, path, for the sake of accuracy and for justice.

### Eyewitness Identification: The Other Forensic Pattern Comparison Discipline.

Eyewitness identification is a widely used forensic pattern comparison tool that has become notorious for its high probability of failure: Misidentifications contribute to about 70% of DNA-confirmed wrongful convictions (13, 45). The eyewitness problem was not tackled in either the 2009 NAS report on forensics or the 2016 PCAST report, but it has been addressed in several critical reviews. These include a 2014 NAS consensus report (46), which was also prepared under the auspices of CSTL by a committee charged with evaluating the underlying science and procedures for collection and use of eyewitness testimony by law enforcement and the courts.

In the practical features of its use, eyewitness identification differs from most other pattern comparison procedures in three respects: 1) The evidence is testimonial; it is not based on any physical artifacts from the crime scene; 2) The comparison is not made between two simultaneously present sensory patterns; it is made between present sensory patterns and a remembered sensory pattern; and 3) The witness is not an “expert” in the sense of certified forensic examiners (though most adults have considerable experience and expertise with facial recognition). Despite these differences, the underlying human information processing task relies on the same brain systems for sensation, perception, cognition, and choice. Understanding of the problem has benefitted greatly from scientific advances in those areas.

Applied eyewitness studies represent, to date, some of the richest injections of modern science into any area of forensic analysis. Most of the recent focus has been on identifying and mitigating factors that affect witness performance (47), as defined by the ability to discriminate a perpetrator from an innocent suspect in a lineup (31) and by identification accuracy (48). While many such factors have been studied, until recently little attention has been paid to the way that individual facial images appear in a lineup. In real casework, methods of lineup presentation have become simplified to an extreme, in part because of resource limitations and because lineups must be constructed ad hoc for every case. With this simplification has come a significant reduction of sensory information that might otherwise be used for identification. To wit, lineups today rarely employ live participants; instead, they employ photographs, which are typically en face and lack visual stereoscopic and motion cues that could reveal three-dimensional (3D) structure. They are absent whole-body information, such as posture and gait, and they are often monochromatic. At the same time, it has become increasingly clear from the basic science of visual object recognition that performance is better when more information-bearing cues are available to the observer (49, 50).

A new study by Heather Flowe and colleagues (51), reported in this collection, takes up this issue of available cues for eyewitness identification using interactive viewing of lineup faces, in which 3D facial images are rotated back and forth at will by the witness. The beauty of this manipulation is that a) the 3D images themselves necessarily provide more cues to inform object recognition, and b) the interactive feature enables a witness to more readily identify and rely upon those cues that are truly “diagnostic,” in the sense that they coincide with memory of the perpetrator but are not shared by all of the lineup faces (52). The empirical result is that the 3D interactive procedure markedly improves, relative to traditional lineups, the ability of eyewitnesses to discriminate perpetrators from innocent suspects, thus reducing the probabilities of misidentification and wrongful conviction. This is a thoughtful example of how good science and new technologies, which are today relatively inexpensive and simple to use, have the potential to transform valued forensic practices and improve the quality of justice.

## The New Threats: Loss of Privacy and Transparency

Forensic investigation is fundamentally about figuring out what happened and who is responsible. The discipline is, in that sense, particularly prone to uses that risk invasion of privacy. A crime scene investigation may turn up evidence that compromises the anonymity—and perhaps also the livelihood, marital welfare, or freedom—of a person having nothing to do with the crime. Recent scientific and technological advances have taken this potential for collateral damage to a new level with the development of highly accurate computer algorithms for identification of patterns in visual images and other forms of data. These algorithms lie at the heart of new surveillance and monitoring systems, such as automated face recognition, that are rapidly growing in use and sophistication. The appeal of this technology is both convenience—paperless border crossings and access to select venues—and security—access restriction and forensic identification of criminal perpetrators (53). But the automated surveillance net also collects information about innocent people and is oftentimes prone to disparate impact (e.g., misidentification of people of color) (54). In a free society, we might trust that information about our private lives is treated with discretion, at least by government actors—including destruction of digital information acquired through broad surveillance and trolling—and that algorithmic bias is recognized and corrected. But without clear regulation and enforcement, these expectations may be aspirational rather than actual, especially given the seductive power and convenience afforded by the tools.

A related concern is the transparency—or lack thereof—associated with the algorithms themselves, which are now being used for a variety of legal applications, such as AI-based pattern comparison (55), and recidivism risk evaluation for decisions about sentencing and parole (56). We include in this collection an essay by Brandon Garrett and Cynthia Rudin (36), a legal scholar and a computer scientist, who make a compelling case for transparency and interpretability of AI-based deciding machines. These authors highlight the fact that there exists a class of algorithms for pattern identification, classification, and prediction that are advertised for their high accuracy performance but are frequently inscrutable, proprietary, or both, meaning that the end user of an algorithm is incapable of articulating how the machine came to a decision that has momentous impact.

Garrett and Rudin make the patent point that in applications for the cause of justice, this failure of algorithmic interpretability can violate rights of discovery, due process, and confrontation and may lead to disparate treatment in violation of rights to equal protection. The authors assert that a “defendant’s constitutional right to confront an adverse testimonial witness cannot be vindicated without the ability to interpret and understand the AI evidence.” Furthermore, “Fairness and discrimination are much easier to assess when models are interpretable.” Yes, of course, this is true from any reasoned scientific perspective. In a just society, shouldn’t an accused be able to confront and interpret the evidence brought to bear against them?

Garrett and Rudin stress that the solution to this problem a) requires recognition by the courts, which are in a position to intervene and b) involves the use of interpretable (“glass box” rather than “black box”) AI, where the underlying measurements and decision criteria are plain to see, inspiring trust in the outcome. This conclusion is powerful, but it prompts a worrisome realization that our widely accepted system for human pattern comparison also largely fails the transparency and interpretability tests. As noted above, the internal values of human-measured pattern similarity and the decision criteria used for classification are neither transparent nor quantified objectively. Moreover, for all the gains of modern neuroscience research, we have today only a limited understanding of how the “human” as an instrument and machine works. What the user of forensic pattern testimony receives is merely a subjective classification decision^**^ and that subjectivity is the principal reason why objective empirical validation is so important.

## The Courts: Gates Installed but Opportunities Missed

The past 100 y have seen major judicial rulings and significant legislative actions designed to ensure that scientific evidence used in litigation is trustworthy. The 1993 *Daubert* ruling assigned trial judges the gatekeeping responsibility to evaluate whether the evidence meets the established standards—empirically tested, peer-reviewed, valid methods, and accurate results—for presentation to the jury. In addition to the points made by Koehler et al. (highlighted above) regarding the effectiveness of the judicial gates (37), we have included three perspectives in this collection [Albright (38), Rakoff and Liu (57), Scurich et al. (29)] that converge on the use of scientific evidence by the courts. Woven together, these articles offer insights into a) the principles and rules for introducing scientific evidence; b) the reasons why our judicial system sometimes fails at the selective admission task; c) the consequences of this failure for efforts to understand scientific truth and administer justice; and d) how we might go about fixing the problem.

### The Rules of Evidence and the Role of the Expert.

In his essay for this collection, Tom Albright offers a “scientist’s perspective” on the use of scientific evidence by the courts (38). This is largely a tutorial for the scientific community on a) the difficult demands placed on the use of scientific evidence in litigation, which are much different from those employed in scientific research, and b) the evidence rules that have been established to guide trial judges in their roles as gatekeepers for admission of evidence into court (6, 58, 59). Echoing a theme raised by Koehler et al. (37), Albright reviews historical variations in the emphasis placed on scientific expert witnesses vs. the scientific knowledge itself. (See also reference (60)). The argument here is that scientific knowledge exists independently of any particular expert. In that spirit, the expert role is best served by good communication, in the form of plain language explanations of scientific knowledge for use by a lay audience. The science itself is freely accessible to anyone for use in the making of practical decisions, as it has been in other applied sciences, such as medicine and engineering. Albright makes the idealist argument that under the intense demands of courtroom litigation, an expert should channel the scientific consensus (“general acceptance”) of the day, for that is the most rational basis for decision given the exigence and resoluteness of the process. As any legal scholar will tell you, however, that idealism runs up hard against the practicalities of our judicial system, including constitutional protection of due process rights. But it is the conceptual standard with which we should start.

The only other native attribute of experts that is of significance is that they represent the “relevant scientific community” (6). Albright notes that the scholarly credentials of experts are valued by juries—often more so, it seems, than the science itself (61). The courts, however, have long struggled to identify the type of expertise that is most relevant to a specific question before the court (62, 63) and to control a veritable circus of experts who have passed the admissibility test (64, 65), often by lack of attention to the relevance standard. We address the latter problem below. Here, we briefly highlight the neglected question of what constitutes relevant science.

Albright argues that the definition of relevance has emerged as a particularly important matter in forensic testimony and deserves greater attention by the judiciary (38). Forensic *practitioners*, who possess expertise in the underlying principles and use of a forensic tool—they know *how* the tool works—have long held sway in trial courts. Scientific *researchers*, by contrast and by definition, possess expertise in experimental design and in the conduct of empirical studies of *how well* a tool or manipulation achieves the desired effect. As decisions from recent cases show (66, 67), it is the researcher who is in the best position to answer what may be the most important question for the trier of fact (63): What is the probability that the forensic testimony is correct?

### A View from the Bench: Courts Armed but not Always Reactive to Scientific Evidence.

Forensic evidence—messy, uncertain, highly subject to bias, and disheartening to pretty much everyone involved—routinely winds its way from crime labs to the courts, where it must first be evaluated, sometimes in a whirlwind of contestable arguments that take place in a *Daubert* admissibility hearing, for credibility by gatekeeping judges. To gain insight into this gatekeeping task from the perspective of the bench, we invited an essay from two prominent judges and influential legal scholars with a longstanding interest in forensic reform (57): Jed Rakoff, senior US District Judge for the Southern District of New York, and Goodwin Liu, Associate Justice of the California Supreme Court (and CSTL alumnus). Rakoff and Liu begin by describing three major developments over the past 30 y that can broadly inform the courts about the validity of forensic evidence: 1) the advent of DNA profiling, which revealed a pervasive problem of wrongful conviction, much of it associated with failures of scientific evidence; 2) the US Supreme Court’s transformative *Daubert* ruling on the validity of scientific evidence (6); and 3) The detailed and high-profile condemnation of the safety and efficacy^††^ of forensic practice by an esteemed scientific organization, the National Academy of Sciences, in their 2009 report.

Rakoff and Liu offer a grim assessment, which is that despite these extraordinary developments, judges are inconsistent, at best, at critical evaluation of forensic evidence that reaches their courts. Some judges adopt a liberal approach to admissibility, rationalized by precedent, and founded on the flimsy premise that bad science will be rejected by juries when experts are subjected to cross-examination (68). Other judges appear to carefully consider the science, appreciating nuances of “relevant scientific community,” “widespread acceptance,” uncertainty of measurement, potential for bias, and manifest at least an implicit understanding of the fragility of our adversarial system for decision-making. In their perspective piece (highlighted in more detail below) Nick Scurich, David Faigman, and Tom Albright echo this concern about inconsistent application, noting that “most judges continue to admit [nonvalidated] forms of forensic evidence without serious scientific review” (29).

### What is the Problem with the Courtroom Gates?

“Why is this?” Rakoff and Liu ask of the inconsistent adoption by courts of carefully considered standards for scientific evidence (57). The authors offer some explanations, the most credible of which is the simple fact that “most judges lack a scientific background—for example, no member of the current Supreme Court holds a degree in science—and do not feel comfortable independently assessing the reliability of scientific evidence.” Scurich et al. go further to say that “[t]his laxity appears to be a dual function of the law’s inertia and ignorance of science.” Inertia reflects the deadening but seemingly immutable role that precedent plays in judicial decisions. Ignorance is the real failure of duty: “*Daubert* sought to impose on judges the responsibility for understanding the empirical grounds on which expert testimony relies.” Regrettably, by the estimation of Scurich et al., “courts have had considerable difficulty employing the *Daubert* factors or Rule 702’s standards” (29).

The practical consequences of failure to exercise discretion over the entry of scientific evidence to the courtroom are huge. Indeed, these are the very reasons that led to legislative and judicial action on evidence admissibility in the first place: Poor quality expert evidence presented to an inexpert trier may lead decisions to be based on opinions that are not sound or true to fact. The common rebuttal is that truth gets sorted out through the adversarial process (69). To illustrate the weakness of this argument and the fragility of courtroom decisions based on unchecked scientific evidence, Albright draws a distinction between generative and terminal adversarial systems for truth-seeking (38). Balanced adversaries in scientific research move forward by generating new experiments that can test the relative merits of their positions—a sort of science playoff round. With each new injection of knowledge, the generative process repeats, ad infinitum, triggered wherever adversarial conflict appears (70). In the courts, this approach is impossible because the adversarial process has a terminal outcome, which instead fosters a decision economy based on competitive marketing and triggers a disruptive cognitive phenomenon in which experts unconsciously adopt opinions about scientific truth that reflect allegiance to the parties that hired them (71). Some compelling solutions to this predictable problem have been proposed, as reviewed by Albright (38), but any step toward implementation faces a minefield of constitutional due process concerns, navigation of which will require greater consilience between the disciplines of science and law. In the meantime, it will still sometimes be true that “the ordinary means successful to aid the jury in getting at the facts, aid, instead of that, in confusing them” (64).

### Improving the Operation of Admissibility Gates for Scientific Evidence.

As explanation for inconsistent application of evidence rules, Rakoff and Liu hypothesize that most judges “do not feel comfortable independently assessing the reliability of scientific evidence.” Indeed, there is a fair and rational case to be made that judges should not, in the first place, be put in the uncomfortable—and risky—position of assuming full responsibility for something that is beyond their ken. As Scurich et al. rightly note in their essay (29), “Courts need more help than *Daubert’s* five generic factors of sound science have so far provided.” Some help does exist. Following the Supreme Court’s 1993 *Daubert* ruling on scientific evidence (6), the Federal Judicial Center (FJC) began publishing the *Reference Manual on Scientific Evidence*, which contains cogent summaries of scientific topics of relevance to the judiciary (e.g., forensics, toxicology, epidemiology). The third edition was published in 2011 jointly by the FJC and the NAS (72), under the auspices of CSTL,^‡‡^ and has become a valued source of information to assist judges with decisions about admissibility. The new Rule 702-2022 may also provide some modest help (73), as it defines a quantitative (and legally typical) standard (“more likely than not”) for determining whether evidence meets the requirements of the rule. But the seriousness of the problem calls for broader strategies for assisting the judiciary on matters of science.

Suggestions made in the past to address the problem of partisan experts [noted above and summarized in the accompanying piece by Albright (38)], which include a science court (74) and ad hoc science consensus panels of the sort commonly used to adjudicate research funding decisions (75), also offer potential tools for judges. A carefully moderated, open, and independent discussion of relevant science by these means, presented to the trial judge in an evidentiary hearing, could go a long way toward overcoming ignorance and ensuring more uniform application of courtroom standards for the quality of scientific evidence.

Another valuable strategy to improve the ability of judges to make sound decisions about admissibility of scientific evidence is proposed by Scurich et al. (29). These authors offer specific guidelines designed to assist judges in evaluating the utility and performance of forensic pattern comparison methods. The guidelines concept is drawn from a highly effective and broadly adopted approach to problems of causal inference in epidemiology. Known today as the “Bradford Hill Guidelines,” in recognition of the developer, Sir Austin Bradford Hill (76), their use is intended to assist physicians in answering key scientific questions about causality—as in, for example, determining whether an observed association between herbicide (e.g., Agent Orange) exposure and prostate cancer reflects a causal relationship.

In law, the Bradford Hill Guidelines for causal inference have nearly verbatim application to torts. While the important question in forensic cases is not directly one of causality—it is about scientific method performance—the same concept with some modification can be used to assist judges in answering key scientific questions that bear on performance. In their essay, Scurich et al. identify and detail four guidelines, which correspond to assessment of 1) plausibility, 2) quality of research design and methods used to assess validity, 3) corroboration, and 4) valid means to generalize from group effects to individual cases. While much of our focus in this *Introduction*, and the focus of the larger reformist literature on forensic practice, has been on the validity of a forensic tool (10, 15), the Scurich et al. approach promotes a much broader view of the performance landscape as a guide for admissibility. The premise is that the results of the four assessment guidelines should not be treated categorically, like a checklist. Rather, they should figure into an estimate of the probability that the proffered testimony is sufficiently trustworthy to be weighed by the trier of fact, which is precisely the goal of an admissibility hearing.

## Conclusions

Forensic practice was born with the noble purpose of seeking justice for people who have been wronged by others. But collection of evidence, measurement, and deductive reasoning alone do not make a science. The grand conceit of forensic pattern comparison disciplines is not simply that a human observer might determine whether two measurements are the same, but whether they are the same according to some reasoned quantitative standard and with known probability of error. To that end, the articles in this *Special Feature* collection convey, in no uncertain terms, that there is a much-needed revolution underway in forensic practice.

Empirical demonstration that conclusions are sometimes wrong and understanding of where the system breaks down are just the beginnings. To become a true science, a forensic pattern discipline must establish an empirical framework for asking the right questions about performance, design studies to assess method validity in precise quantitative terms, and appreciate the operating characteristics of the forensic instrument employed—the human brain—and its high susceptibility to bias under conditions of uncertainty.

The goal of a true forensic science is not just to decide, but to understand the legitimacy of human decisions in the messy and consequential world of evidence. Used with recognition of the potential for bias, and applied with transparency and respect for privacy, discoveries in the sciences of human information processing and advances in statistics and modeling can help achieve this goal. But getting forensic decisions right is only half the problem; the judiciary must also be educated and responsive to its gatekeeping mandate. Though all of this transpires within the arena of law, these are not matters that should be left exclusively to law enforcement and the courts. Progress made thus far toward a “scientific re-invention of forensic science” is fruit of a larger science-law consilience with many benefits for justice. Much of this consilience has emerged naturally from interdisciplinary pursuits, such as the NAS Committee on Science, Technology, and Law, and forensic initiatives at the American Association for the Advancement of Science. The forensic revolution is not over, far from it. But the fact that it has begun at all and its successes thus far are testament to the critical importance of interdisciplinary work at the larger interface between scientific knowledge and legal policy.

## Footnotes

^*^Because forensic practice incorporates elements of the scientific method, such as data acquisition and deductive reasoning, the discipline is often called “forensic science.”

^†^To be sure, adequate proficiency testing can contribute to knowledge about accuracy even if the examiner is, in some sense, the "instrument." However, adequate and rigorous proficiency testing has not been a general feature of the pattern identification fields.

^‡^Text excerpted from 2017 AAAS interview with Jed Rakoff, US District Judge for the Southern District of New York. It was intended as a caricature of experience claims, not actual testimony. See also ref. 77.

^§^The primary recommendation of the NAS report was to establish a new federal entity—a National Institute of Forensic Science—but congressional legislation to this end did not advance.

^¶^As called for in the 2009 NAS report on forensics (10).

^#^This phrase has been a frequent component of legal testimony for decades. In 2016, the National Commission on Forensic Science approved a recommendation to the DOJ to adopt new guidelines that discourage use of the phrase, in view of its lack of substantive meaning. The Attorney General accepted the recommendation and issued a memorandum to effect relevant policies and guidelines.

^‖^See the 2020 DOJ’s disturbing lack of insight into this matter (78).

^**^A subjective decision is an observer-dependent decision. An argument can be made that to the extent that a decision is made by uninterpretable AI, that decision is observer dependent and subjective.

^††^By analogy to clinical trials in medicine, safety refers to the extent to which the tool yields no dangerous side effects, such as wrongful conviction. Efficacy, in turn refers to the extent to which the method is sensitive enough to identify the culprit.

^‡‡^A fourth edition is in preparation, as of this writing.

## References

[1] W. Van Winkle, R. P. Herwick, H. O. Calvery, A. Smith, Laboratory and clinical appraisal of new drugs. J. Am. Med. Assoc. **126**, 958–961 (1944).

[2] Drug Amendments of 1962, Public Law 87-781, https://www.govinfo.gov/content/pkg/STATUTE-76/pdf/STATUTE-76-Pg780.pdf. Accessed 21 September 2023.

[3] FDA, Regulations relating to good clinical practice and clinical trials. https://www.fda.gov/about-fda/center-drug-evaluation-and-research-cder/fda-regulations-relating-good-clinical-practice-and-clinical-trials. Accessed 7 August 2023.

[4] Anonymous Note, Lobotomy: Surgery for the insane. Stanford Law Rev. **1**, 463–474 (1949), 10.2307/1226372.

[5] G. J. Annas, L. H. Glantz, Psychosurgery: The law’s response. BUL Rev. **54**, 249 (1974). 11661084

[6] *Daubert v. Merrell Dow Pharmaceuticals Inc.*, 509 U.S. 579 (1993).

[7] *Kumho Tire Co. v Carmichael*, 526 U.S. 137 (1999).

[8] *General Electric Co. v Joiner*, 522 U.S. 136 (1997).

[9] J. H. Skene, Up to the courts: Managing forensic testimony with limited scientific validity. Judicature **102**, 39 (2018).

[10] National Research Council, Strengthening Forensic Science in the United States: A Path Forward (National Academies Press, 2009).

[11] J. L. Mnookin, S. A. Cole, I. E. Dror, B. A. Fisher, The need for a research culture in the forensic sciences. UCLA L. Rev. **58**, 725–779 (2011).

[12] J. L. Mnookin, Fingerprint evidence in the age of DNA profiling. Brooklyn L. Rev. **67**, 13–70 (2001).

[13] B. L. Garrett, Convicting the Innocent: Where Criminal Prosecutions Go Wrong (Harvard University Press, 2011).

[14] National Commission on Forensic Science, Reflecting Back— Looking Toward the Future (National Commission on Forensic Science, 2017). https://www.justice.gov/archives/ncfs/page/file/959356/download. Accessed 21 September 2023.

[15] President’s Council of Advisors on Science and Technology, Executive Office of the President of the United States, Report to the president: Forensic science in criminal courts: Ensuring scientific validity of feature-comparison methods. https://obamawhitehouse.archives.gov/sites/default/files/microsites/ostp/PCAST/pcast_forensic_science_report_final.pdf. Accessed 21 September 2023.

[16] P. Stout, The secret life of crime labs. Proc. Natl. Acad. Sci. U.S.A. **120**, e2303592120 (2023). 37782808 10.1073/pnas.2303592120 PMC10576105

[17] R. Blumenthal, Officials ignored Houston lab’s troubles, report finds. NY Times (2005), https://www.nytimes.com/2005/07/01/us/officials-ignored-houston-labs-troubles-report-finds.html. Accessed 21 September 2023.

[18] M. R. Bromowich, Final report of the independent investigator for the Houston Police Department Crime Laboratory and Property Room (2007). http://www.hpdlabinvestigation.org/reports/070613report.pdf. Accessed 21 September 2023.

[19] M. Pitcher, How Peter Stout turned around Houston’s crime lab. Texas Observer (2022). https://www.texasobserver.org/peter-stout-houston-crime-lab-forensic-science-center/. Accessed 21 September 2023.

[20] B. L. Garrett, Autopsy of a Crime Lab: Exposing the Flaws in Forensics (University of California Press, 2021).

[21] I. E. Dror, A. E. Peron, S. L. Hind, D. Charlton, When emotions get the better of us: The effect of contextual top-down processing on matching fingerprints. Appl. Cogn. Psychol. **19**, 799–809 (2005).

[22] National Commission on Forensic Science, Views of the Commission: Ensuring that forensic analysis is based upon task-relevant information (2015). https://www.justice.gov/archives/ncfs/file/818196/download. Accessed 21 September 2023.

[23] W. R. Oliver, J. Fudenberg, J. A. Howe, L. C. Thomas, Cognitive bias in medicolegal death investigation. Acad. Forensic Pathol. **5**, 548–560 (2015).

[24] W. R. Oliver, Comment on Dror, Kukucka, Kassin, and Zapf (2018), “When expert decision making goes wrong”. J. Appl. Res. Memory Cogn. **7**, 314–315 (2018).

[25] W. R. Oliver, Comment on Kukucka, Kassin, Zapf, and Dror (2017), “Cognitive bias and blindness: A global survey of forensic science examiners”. J. Appl. Res. Memory Cogn. **7**, 161 (2018).

[26] W. C. Thompson, Shifting decision thresholds can undermine the probative value and legal utility of forensic pattern-matching evidence. Proc. Natl. Acad. Sci. U.S.A. **120**, e2301844120 (2023). 37782790 10.1073/pnas.2301844120 PMC10576151

[27] National Institute of Justice (2022) The Slow but Steady March Towards a More Reliable Forensic Science. https://nij.ojp.gov/topics/articles/slow-steady-march-towards-more-reliable-forensic-science. Accessed 21 September 2023.

[28] United States v Garsson, 291 F. 646 (S.D. N.Y., 1923).

[29] N. Scurich, D. L. Faigman, T. D. Albright, Scientific guidelines for evaluating the validity of forensic feature-comparison methods. Proc. Natl. Acad. Sci. U.S.A. **120**, e2301843120 (2023). 37782809 10.1073/pnas.2301843120 PMC10576079

[30] J. Coen, E. Coen, The Big Lebowski (Gramercy Pictures (I), 1998).

[31] L. Mickes, H. D. Flowe, J. T. Wixted, Receiver operating characteristic analysis of eyewitness memory: Comparing the diagnostic accuracy of simultaneous versus sequential lineups. J. Exp. Psychol. Appl. **18**, 361–376 (2012). 23294282 10.1037/a0030609

[32] S. Gepshtein, Y. Wang, F. He, D. Diep, T. D. Albright, A perceptual scaling approach to eyewitness identification. Nat. Commun. **11**, 3380 (2020). 32665586 10.1038/s41467-020-17194-5 PMC7360747

[33] V. L. Phillips, M. J. Saks, J. L. Peterson, The application of signal detection theory to decision-making in forensic science. J. Forensic Sci. **46**, 294–308 (2001). 11305431

[34] T. D. Albright, How to make better forensic decisions. Proc. Natl. Acad. Sci. U.S.A. **119**, e2206567119 (2022). 36099301 10.1073/pnas.2206567119 PMC9499562

[35] M. B. Thompson, J. M. Tangen, D. J. McCarthy, Expertise in fingerprint identification. J. Forensic Sci. **58**, 1519–1530 (2013). 23786258 10.1111/1556-4029.12203

[36] B. L. Garrett, C. Rudin, Interpretable algorithmic forensics. Proc. Natl. Acad. Sci. U.S.A. **120**, e2301842120 (2023). 37782786 10.1073/pnas.2301842120 PMC10576126

[37] J. J. Koehler, J. L. Mnookin, M. J. Saks, The scientific reinvention of forensic science. Proc. Natl. Acad. Sci. U.S.A. **120**, e2301840120 (2023). 37782789 10.1073/pnas.2301840120 PMC10576124

[38] T. D. Albright, A scientist’s take on scientific evidence in the courtroom. Proc. Natl. Acad. Sci. U.S.A. **120**, e2301839120 (2023). 37782801 10.1073/pnas.2301839120 PMC10576137

[39] Y. Sterchi, N. Hättenschwiler, A. Schwaninger, Detection measures for visual inspection of X-ray images of passenger baggage. Atten., Percept., Psychophys. **81**, 1297–1311 (2019). 30684203 10.3758/s13414-018-01654-8 PMC6647488

[40] P. C. Brennan , Radiologists can detect the ‘gist’ of breast cancer before any overt signs of cancer appear. Sci. Rep. **8**, 8717 (2018). 29880817 10.1038/s41598-018-26100-5 PMC5992208

[41] S. Waite , Analysis of perceptual expertise in radiology—Current knowledge and a new perspective. Front. Hum. Neurosci. **13**, 213 (2019). 31293407 10.3389/fnhum.2019.00213 PMC6603246

[42] T. Gneiting, A. E. Raftery, Weather forecasting with ensemble methods. Science **310**, 248–249 (2005). 16224011 10.1126/science.1115255

[43] I. van Hagen, Flying the F-35 stealth fighter can leave pilots looking “like they are 100 years old”, says test pilot. Business Insider (2023). https://www.businessinsider.com/f-35-test-pilots-intense-flying-top-us-fighter-jet-2023-6. Accessed 10 July 2023.

[44] S. Halpern, The rise of A.I. fighter pilots. The New Yorker (2022). https://www.newyorker.com/magazine/2022/01/24/the-rise-of-ai-fighter-pilots. Accessed 10 July 2023.

[45] B. L. Garrett, Judging innocence. Columbia Law Rev. **108**, 55–142 (2008).

[46] National Research Council, Identifying the Culprit: Assessing Eyewitness Identification (National Academies Press, 2015).

[47] G. L. Wells, Applied eyewitness-testimony research: System variables and estimator variables. J. Pers. Soc. Psychol. **36**, 1546 (1978).

[48] L. Mickes, Receiver operating characteristic analysis and confidence–accuracy characteristic analysis in investigations of system variables and estimator variables that affect eyewitness memory. J. Appl. Res. Memory Cogn. **4**, 93 (2015).

[49] G. Meinhardt, M. Persike, B. Mesenholl, C. Hagemann, Cue combination in a combined feature contrast detection and figure identification task. Vision Res. **46**, 3977–3993 (2006). 16962156 10.1016/j.visres.2006.07.009

[50] T. P. Saarela, M. S. Landy, Integration trumps selection in object recognition. Curr. Biol. **25**, 920–927 (2015). 25802154 10.1016/j.cub.2015.01.068 PMC4382589

[51] M. Meyer , Enabling witnesses to actively explore faces and reinstate study-test pose during a lineup increases discriminability. Proc. Natl. Acad. Sci. U.S.A. **120**, e2301845120 (2023). 37782811 10.1073/pnas.2301845120 PMC10576112

[52] J. T. Wixted, L. Mickes, A signal-detection-based diagnostic-feature-detection model of eyewitness identification. Psychol. Rev. **121**, 262 (2014). 24730600 10.1037/a0035940

[53] D. Harwell, C. Timberg, How America‘s surveillance networks helped the FBI catch the Capitol mob. The Washington Post (2021).

[54] P. Grother, M. Ngan, K. Hanaoka, Face Recognition Vendor Test (FRVT): Part 3, Demographic Effects (National Institute of Standards and Technology, Gaithersburg, MD, 2019).

[55] S. Barrington, H. Farid, A comparative analysis of human and AI performance in forensic estimation of physical attributes. Sci. Rep. **13**, 4784 (2023). 36959267 10.1038/s41598-023-31821-3 PMC10036317

[56] K. B. Forrest, When Machines Can be Judge, Jury, and Executioner: Justice in the Age of Artificial Intelligence (World Scientific, 2021).

[57] J. S. Rakoff, G. Liu, Forensic science: A judicial perspective. Proc. Natl. Acad. Sci. U.S.A. **120**, e2301838120 (2023). 37782784 10.1073/pnas.2301838120 PMC10576125

[58] Frye v. United States, 293 F. 1013 (D.C. Cir., 1923).

[59] Federal Rules of Evidence, Rule 702, https://www.rulesofevidence.org/article-vii/rule-702/. Accessed 21 September 2023.

[60] J. L. Mnookin, Expert evidence, partisanship, and epistemic competence. Brooklyn Law Rev. **73**, 1009 (2007).

[61] J. J. Koehler, N. J. Schweitzer, M. J. Saks, D. E. McQuiston, Science, technology, or the expert witness: What influences jurors’ judgments about forensic science testimony? Psychol., Public Policy, Law **22**, 401 (2016).

[62] E. Biber, Which science? Whose science? How scientific disciplines can shape environmental law. Univ. Chicago Law Rev. **79**, 471–552 (2012).

[63] D. L. Faigman, N. Scurich, T. D. Albright, The need for anti-expert experts to rebut claims of junk forensic science. Scientific American (2022).

[64] L. Hand, Historical and practical considerations regarding expert testimony. Harvard Law Rev. **15**, 40–58 (1901).

[65] P. D. Carrington, T. L. Jones, Reluctant experts. Law Contemp. Prob. **59**, 51–65 (1996).

[66] R. Balko, Devil in the grooves: The case against forensic firearms analysis. The Watch (2023). https://radleybalko.substack.com/p/devil-in-the-grooves-the-case-against. Accessed 21 September 2023.

[67] *State of Illinois v Ricky Winfield* (Case no. # 15 CR 14066-01).

[68] P. J. Neufeld, The (near) irrelevance of *Daubert* to criminal justice and some suggestions for reform. Am. J. Public Health **95**, S107–S113 (2005). 16030325 10.2105/AJPH.2004.056333

[69] *Boucher v U.S. Suzuki Motor Corp.*, 73 F.3d 18 (2d Cir., 1996)

[70] K. Popper, The Logic of Scientific Discovery (Basic Books, New York, NY, 1959).

[71] D. C. Murrie, M. T. Boccaccini, Adversarial allegiance among expert witnesses. Annu. Rev. Law Soc. Sci. **11**, 37–55 (2015).

[72] National Research Council, Reference Manual on Scientific Evidence (National Academies Press, 2011). 41955395

[73] Supreme Court 2023 order to amend Federal Rules of Evidence. https://www.supremecourt.gov/orders/courtorders/frev23_5468.pdf. Accessed 21 September 2023.

[74] A. Kantrowitz, The science court experiment. Jurimetrics J. **17**, 332–341 (1977).

[75] S. Perry, The NIH consensus development program. New Engl. J. Med. **317**, 485–488 (1987). 3614293 10.1056/NEJM198708203170806

[76] A. B. Hill, The environment and disease: Association or causation? Proc. R. Soc. Med. **58**, 295–300 (1965). 14283879 10.1177/003591576505800503 PMC1898525

[77] D. J. Capra, Symposium on forensic expert testimony, Daubert, and Rule 702. Fordham L. Rev. **86**, 1459–1550 (2017).

[78] T. D. Albright, The US Department of Justice stumbles on visual perception. Proc. Natl. Acad. Sci. U.S.A. **118**, e2102702118 (2021). 34031260 10.1073/pnas.2102702118 PMC8214698

